# Supplementary material for: A randomized trial in a massive online open course shows people don’t know what a statistically significant relationship looks like, but they can learn
Source: PeerJ. 2014 Oct 16;2:e589. doi: 10.7717/peerj.589 (PMC4203023; doi:10.7717/peerj.589)
Supplement: Supplemental Information [file peerj-02-589-s001.pdf]

Supplemental Materials

Aaron Fisher, G. Brooke Anderson, Roger Peng, Jeff Leek*

August 25, 2014

Contents

1 Coursera Background Information **1**

2 Previous Quiz Questions on P-values **2**

3 Example Plots Shown to Users **3**

4 Outlier Plot Category **7**

5 Models Used in Analysis **8**

6 Link to Code **10**

1 Coursera Background Information

Coursera is an online learning platform providing massive courses to over 5 million people, as of 2013 [1]. Subjects offered include statistics, programming, chemistry, physics, engineering, and education [2]. Participation in courses is free and voluntary, although a fee is charged if a student seeks a certificate of completion or, in the few courses for which it is offered, college credit for the course [3]. This voluntary learning platform therefore creates an intriguing opportunity to perform trials for evidence-based education, as courses collect large numbers of students who are voluntarily participating in courses, most for the sole purpose of learning and with no penalty for poor course performance.

Although attrition rates from MOOCs can be much higher than from traditional courses [4, 5], the massive size of these courses means even with high attrition the number of students can still be much higher than in traditional courses, offering the opportunity for statistically powerful observational and experimental research [6]. The US Department of Education recognizes the opportunity Coursera courses and other MOOCs offer exciting opportunities for research on learning and cognition, and recommends that, “Institutional Review Board (IRB) documentation and approval processes for research involving digital learning systems and resources that carry minimal risk should be streamlined to accelerate their development without compromising needed rights and privacy protections” [7]. In particular, they highlight that “new technologies can capture, organize, and analyze vast quantities of data,” while “in the recent past, data on learning had to be laboriously and slowly collected, and consequently, data were scarce” and that

*Correspondence to: jleek@jhsph.edu

"they provide an opportunity to conduct controlled random-assignment experiments [8] much more rapidly than was previously possible" [7, 8]. Finally, online learning platforms offer the opportunity to perform randomized experiments while offering students educational assessments with rapid feedback, so that "students are learning about a concept or how to execute a skill at the same time the system is attempting to gauge their competence in that knowledge or skill" [7].

Despite the relatively short history of MOOCs, some studies have been done using data generated from these courses or surveys administered to course participants [9]. Studies include investigations of the effectiveness of mastery learning [10, 11], attrition rates during courses [4], and a comparison of the use of blogs versus forums by students [12].

2 Previous Quiz Questions on P-values

Before taking the survey, students were exposed to two quiz questions relating to P-values. These questions are presented below.

Question 1

	$\beta = 0$	$\beta \neq 0$	CLAIMS TOTALS
Claim $\beta = 0$	50	10	60
Claim $\beta \neq 0$	5	20	25
Hypothesis Totals	55	30	85

What is the (observed) rate of false discoveries? What is the (observed) rate of false positives?

Question 2

Generate P-values according to the following code:

```
set.seed(3343)
pValues = rep(NA,100)
for(i in 1:100){
  z = rnorm(20)
  x = rnorm(20)
  y = rnorm(20,mean=0.5*x)
  pValues[i] = summary(lm(y ~ x))$coef[2,4]
}
```

How many are significant at the alpha = 0.1 level when controlling the family wise error rate using the methods described in the lectures? When controlling the false discovery rate at the alpha = 0.1 level as described in the lectures?

3 Example Plots Shown to Users

All plots shown to users were selected from a library of 80 pregenerated plots. This full library is available at

`https://github.com/aaronjfisher/visual_pvalue/tree/master`

On each attempt of the survey, users were shown eight plots from seven different categories. More specifically, two plots were shown from the reference category (of which one was significant and one was not) and one plot was shown from each of the remaining categories (each randomly chosen to be either significant or non-significant). Users were also shown a plot with an added outlying data point, but responses to plots in this “Outlier” category were not directly compared against responses to other plot categories in our final analysis (see Section 4). Overall, on each attempt of the survey, users were shown nine plots from a total of eight different categories. For each of these plot categories, Figures 1, 2, 3, and 4 show two examples from our pregenerated library. The plot titles for these figures contain the P-value for the underlying relationship shown. In the survey shown to users, plots were generally simply titled “Sample Data.” The exceptions to this rule were plots containing best fit lines or lowess curves, which were titled “Sample Data with OLS Best Fit Line,” or “Sample Data with Lowess Line,” respectively. The plots in Figures 1, 2, 3, and 4 with blue highlighted borders collectively represent one complete set of plots that a survey user might have seen. Note that this set of highlighted plots contains two plots from the reference category, one significant and one not significant, and one plot from each of the remaining categories.

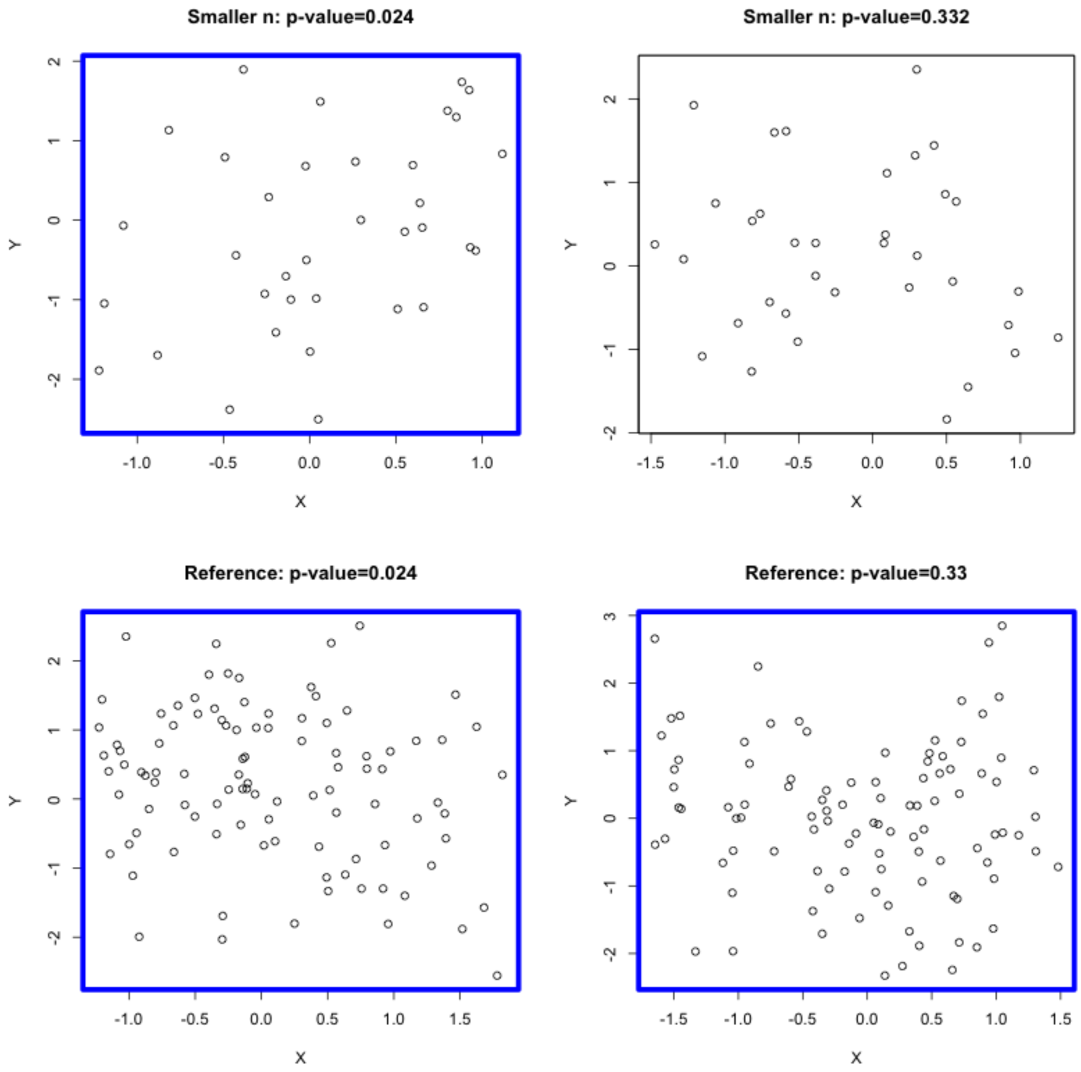

Figure 1: Example Plots Shown to Users (Part 1) - Example plots from the "Lower n" and "Reference" catogories. Plots on the left are significant, and plots on the right are nonsignificant. The plots with blue highlighted borders collectively represent one complete set of plots that a survey user might have seen.

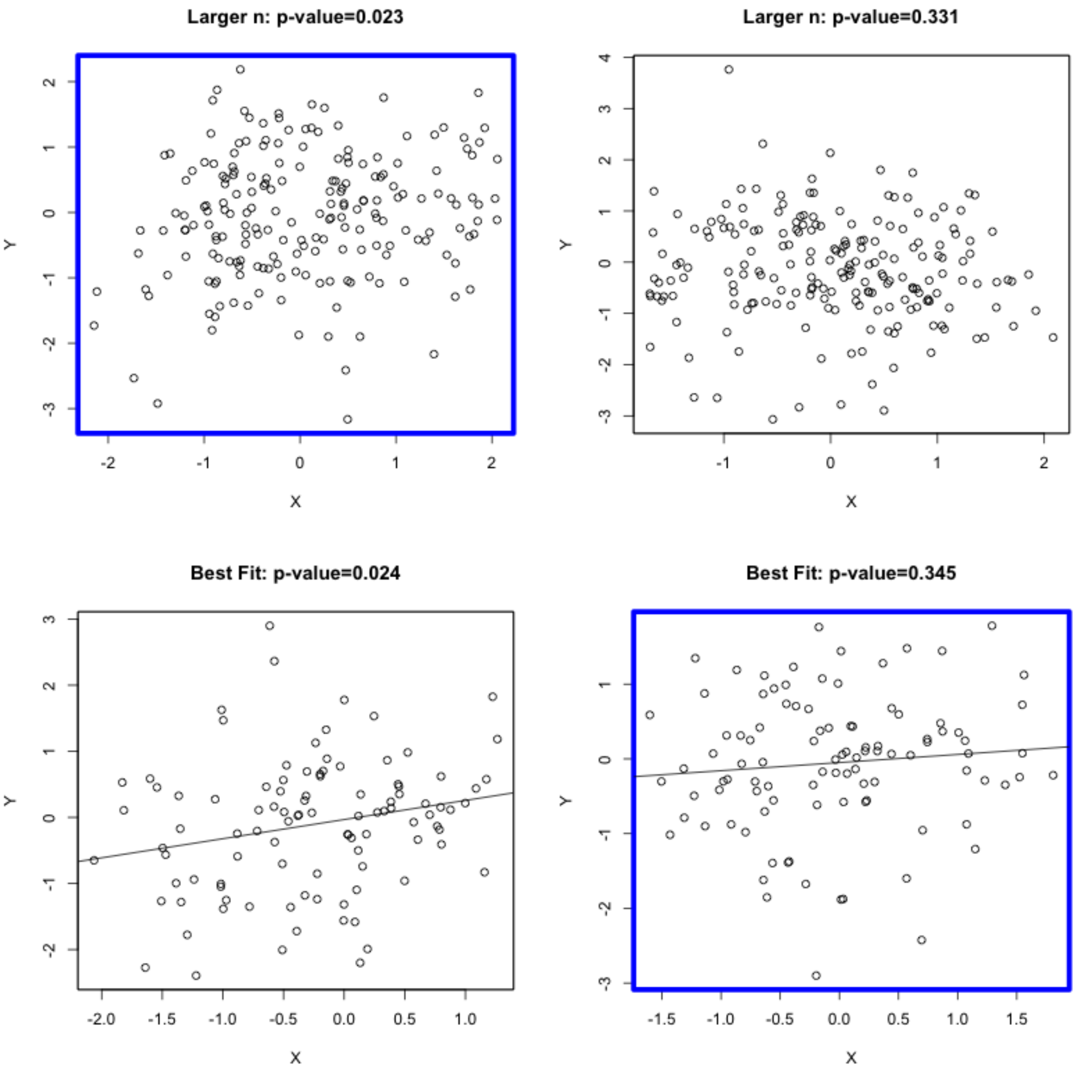

Figure 2: Example Plots Shown to Users (Part 2) - Example plots from the “Larger n” and “Best Fit” catogories. The plots with blue highlighted borders collectively represent one complete set of plots that a survey user might have seen.

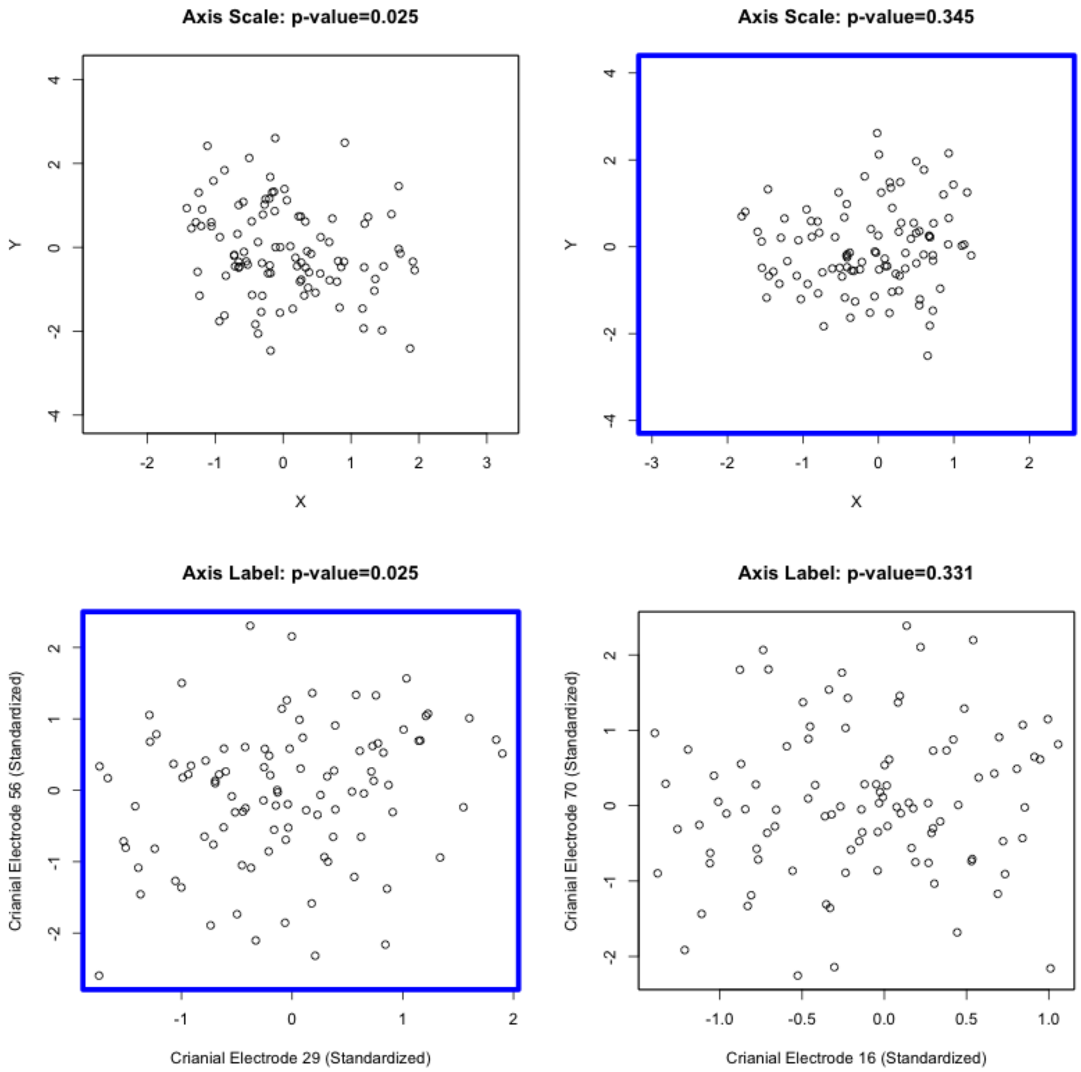

Figure 3: Example Plots Shown to Users (Part 3) - Example plots from the "Axis Label" and "Axis Scale" catogories. The plots with blue highlighted borders collectively represent one complete set of plots that a survey user might have seen.

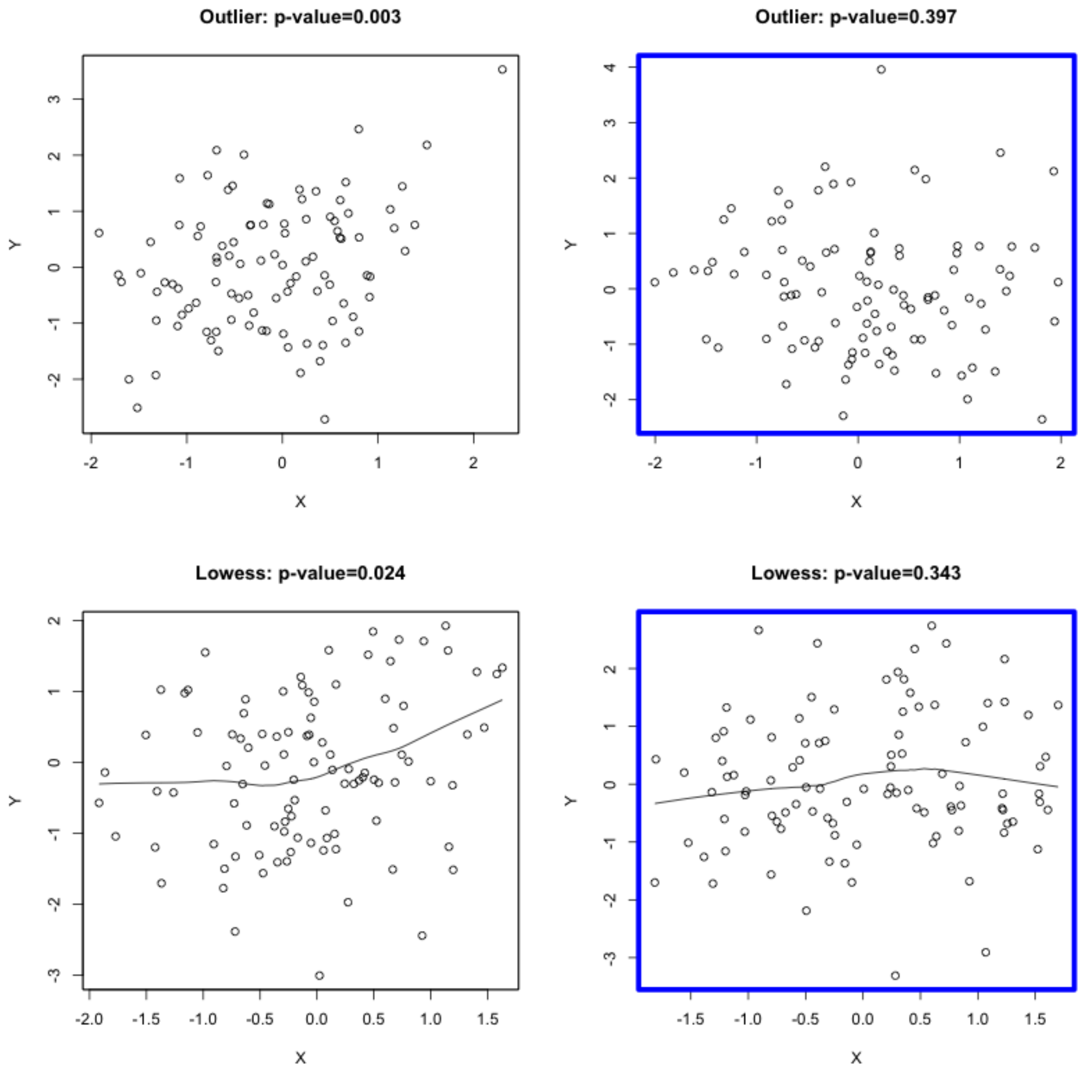

Figure 4: Example Plots Shown to Users (Part 4) - Example plots from the “Outlier” and “Lowess” catogories. The plots with blue highlighted borders collectively represent one complete set of plots that a survey user might have seen.

4 Outlier Plot Category

One of the presentation styles shown to users was an “Outlier” plot category (See top panel of Figure 4). Our goal in showing users this plot category was to see how the addition of outlier points affected user accuracy in classifying statistical significance. However, we neglected to account for the fact that changing the position of the points not only created a visual outlier, but also changed the underlying P-value. The plots created for this “Outlier” category showed relationships with P-values either below 0.005, or above 0.36, all of which were outside the range

of P-values for the other plot categories. When comparing these outlier plots against the reference category plots, it is not possible to separate the visual effect of an added outlier from the visual effect of a different underlying significance level. As such, estimates for the effect of adding an outlier are not included in the main body of our report.

The procedure used to generate significant "Outlier" category plots consisted of first generating a standard sample with a P-value between 0.023 and 0.025. The central datapoint of the sample was then moved to one of the corners of the plot, creating an outlier. If the slope of the generated standard plot was negative, the central point was moved to be 1 standard deviation to the left of the point with the lowest X coordinate, and one standard deviation above the point with the highest Y coordinate. If the slope of the generated standard plot was positive, the central point was moved to be one standard deviation to the right of the highest X coordinate, and one standard deviation above the highest Y coordinate.

The procedure for generating nonsignificant outlier plots consisted of first generating a standard sample with a P-value between 0.33 and 0.35. The central datapoint of the sample was then selected, and its Y coordinate was changed to be $\sqrt{2}$ standard deviations above the largest Y coordinate in the plot. The X coordinate of this outlier point was left unchanged.

5 Models Used in Analysis

In all of our analyses, we created separate models for the probability of correctly classifying significant plots, and for the probability of correctly classifying nonsignificant plots. We refer to these two accuracy metrics respectively as the "human sensitivity to significance," and "human specificity to nonsignificance." Both accuracy metrics were modeled modeled using logistic regressions with random intercept terms, and were fit using the lme4 package in R.

Our first aim was to estimate the baseline sensitivity and specificity, and to analyze differences in these accuracy metrics across plot categories. To address this aim, we looked only at users' first attempts of the survey. Let Y_{ij} be the indicator that user i correctly classified the significance of the jth plot they were shown. To estimate sensitivity, we fit the following logistic regression model on data where the plots shown to users depicted significant relationships

$$\begin{aligned} Y_{ij} &\sim \text{Bernoulli}(\text{Sensitivity}_{ij}) \\ \text{Sensitivity}_{ij} &= \text{logit}^{-1}\left(b_{0i} + \beta_1 + \sum_{k=2}^{K} \beta_k I(\text{category}_{ij} = k)\right) \\ b_{0i} &\sim N(0, \sigma_b^2) \end{aligned}$$

Where β_1 is the logit of sensitivity in the "Reference" plot category, K is the total number of plot categories shown to users, including the reference category ($K = 8$), β_k is a fixed effect representing the change in the logit of sensitivity for the kth plot category, relative to the "Reference" category, $I(\text{category}_{ij} = k)$ is the indicator that the jth plot shown to user i was in the kth category, and the b_{0i} terms represent user-specific random intercepts. This model was fit on a total of 9063 answer submissions.

To estimate specificity for each plot category, we fit the same model, but only on data where the plots shown to users depicted nonsignificant relationships

$$
\begin{aligned}
Y_{ij} &\sim \text{Bernoulli}(\text{Specificity}_{ij}) \\
\text{Specificity}_{ij} &= \text{logit}^{-1}\left(a_{0i} + \alpha_1 + \sum_{k=2}^{K} \alpha_k I(\text{category}_{ij} = k)\right) \\
a_{0i} &\sim N(0, \sigma_a^2)
\end{aligned}
$$

Here, the a_{0i} terms are user-specific random intercepts, α_1 is the logit of specificity in the "Reference" category, and the α_k represent the change in the logit of specificity for the kth category, relative to the "Reference" category. This model was fit on a total of 9032 answer submissions.

Our second aim was to estimate learning effects by comparing sensitivity and specificity across first and second attempts of the survery. For this analysis, we only used data from users who submitted the survery more than once. Of these students, 92% completed their first attempt of the survey, and 99% completed their second attempt of the survey. Plots shown on each attempt of the survey were randomly and independently drawn from our library of pregenerated plots, which resulted in some users seeing the same exact plot more than once, across multiple attempts of the survey. These duplicate responses to the same plots comprised 12.8% of second attempt questions, and were discarded in this analysis.

Again, we fit two separate logistic regression models, one for sensitivity and one for specificity. Both models set accuracy as a function of plot category and attempt number. Random intercepts were again used to account for user-specific subjective thresholds for declaring significance.

To estimate learning effects regarding sensitivity, we fit the model:

$$
\begin{aligned}
Y_{ij} &\sim \text{Bernoulli}(\text{Sensitivity}_{ij}) \\
\text{Sensitivity}_{ij} &= \text{logit}^{-1}\left(b_{0i} + \beta_1 + \lambda_1 I(\text{Attempt}_{ij} = 2) + \sum_{k=2}^{K} I(\text{category}_{ij} = k)(\beta_k + \lambda_k I(\text{Attempt}_{ij} = 2))\right) \\
b_{0i} &\sim N(0, \sigma_b^2)
\end{aligned}
$$

Where $I(\text{Attempt}_{ij} = 2)$ is the indicator that the jth plot shown to user i was on that user's second attempt of the survey, and the λ_k represents change in logit of sensitivity in the second attempt of the survey for the kth plot category. This model was fit on a total of 846 answer submissions.

To estimate learning effects regarding specificity, we fit the model:

$$
\begin{aligned}
Y_{ij} &\sim \text{Bernoulli}(\text{Specificity}_{ij}) \\
\text{Specificity}_{ij} &= \text{logit}^{-1}\left(a_{0i} + \alpha_1 + \delta_1 I(\text{Attempt}_{ij} = 2) + \sum_{k=2}^{K} I(\text{category}_{ij} = k)(\alpha_k + \delta_k I(\text{Attempt}_{ij} = 2))\right) \\
a_{0i} &\sim N(0, \sigma_a^2)
\end{aligned}
$$

Where the δ_k represents change in the logit of specificity on the second attempt of the survey for the kth plot category. This model was fit on a total of 859 answer submissions.

As discussed in Section 4, the plots show to users which had added outliers also had underlying P-values that were incomparable to those in the reference category. For the purposes of better estimating the random intercept terms (b_{0i} and a_{0i}), the data from these "Outlier" plots was still

included when fitting the four models described in this section. However, the fixed coefficients corresponding accuracy in the "Outlier" category were not directly interpreted.

Part of the variation in users first response to the survey is due to latent differences in the personal, subjective thresholds each student has for declaring a relationship to be significant. In the above models, we use person-specific random intercepts to account for these thresholds. For the models analyzing first attempts of the survey, these random intercepts explain 10.8% of the latent variation in sensitivity, and 15.1% of the variation in specificity. In our models used to analyze differences between the first and second attempts of the survey, user-specific random intercept terms explained 4.2% of the variation in sensitivity, and 12.0% of the variation in specificity.

6 Link to Code

Additional supplementary materitals, including the code used to analyze the survey results, is available at

`https://github.com/aaronjfisher/visual_pvalue/tree/master`

References

[1] Coursera. (2013) A Triple Milestone: 107 Partners, 532 Courses, 5.2 Million Students and Counting! `http://blog.coursera.org/post/64907189712/a-triple-milestone-107-partners-532-courses-5-2` (accessed August 21, 2014).

[2] Coursera. (2014). `https://www.coursera.org/courses` (accessed August 21, 2014).

[3] Gordon, L. (2013) Four Coursera Online Classes are Deemed Worthy of College Credit. *Los Angeles Times.* `http://articles.latimes.com/2013/feb/07/local/la-me-0207-online-credit-20130207` (accessed August 21, 2014).

[4] Clow, D. (2013) MOOCs and the Funnel of Participation. *Proceedings of the Third International Conference on Learning Analytics and Knowledge (Association for Computing Machinery)* pp. 185–189.

[5] Marcus, J. (2013) All Hail MOOCs! Just Don't Ask if They Actually Work. *Time Magazine.* `http://nation.time.com/2013/09/12/all-hail-moocs-just-dont-ask-if-they-actually-work/` (accessed August 21, 2014).

[6] Parry, M. (2012) 5 Ways that edX Could Change Education. *The Chronicle of Higher Education.*

[7] U.S. Department of Education, Office of Educational Technology. (2013) Expanding Evidence Approaches for Learning in a Digital World, (U.S. Department of Education, Office of Educational Technology), Technical report. `http://www.ed.gov/edblogs/technology/files/2013/02/Expanding-Evidence-Approaches.pdf` (accessed August 21, 2014).

[8] Shadish, W. R & Cook, T. D. (2009) The Renaissance of Field Experimentation in Evaluating Interventions. *Annual Review of Psychology* **60**, 607–629.

[9] Liyanagunawardena, T. R, Adams, A. A, & Williams, S. A. (2013) MOOCs: A Systematic Study of the Published Literature 2008-2012. *The International Review of Research in Open and Distance Learning* **14**, 202–227.

[10] Do, C. B, Chen, Z, Brandman, R, & Koller, D. (2013) Self-Driven Mastery in Massive Open Online Courses. *MOOCs FORUM* **1**, 14–16.

[11] Pardos, Z. A, Bergner, Y, Seaton, D. T, & Pritchard, D. E. (2013) Adapting Bayesian Knowledge Tracing to a Massive Open Online Course in edX. *Proceedings of the 6th International Conference on Educational Data Mining.* `http://www.educationaldatamining.org/IEDMS/EDM2013` (accessed August 21, 2014).

[12] Mak, S, Williams, R, & Mackness, J. (2010) Blogs and Forums as Communication and Learning Tools in a MOOC. *University of Lancaster.*
